# Evidence of H10N8 influenza virus infection among swine in southern China and its infectivity and transmissibility in swine

**DOI:** 10.1080/22221751.2019.1708811

**Published:** 2020-01-03

**Authors:** Xinliang Fu, Yunmao Huang, Bo Fang, Yixing Liu, Mengkai Cai, Ruting Zhong, Junming Huang, Qi Wenbao, Yunbo Tian, Guihong Zhang

**Affiliations:** a College of Veterinary Medicine, South China Agricultural University, Guangzhou, People’s Republic of China; b Key laboratory of Zoonosis Prevention and Control of Guangdong Province, Guangzhou, People’s Republic of China; c College of Animal Science & Technology, Zhongkai University of Agriculture and Engineering, Guangzhou, People’s Republic of China; d Guangdong Province Key Laboratory of Waterfowl Healthy Breeding, Guangzhou, People’s Republic of China

**Keywords:** H10N8, influenza virus, infectivity, pathogenicity, transmissibility

## Abstract

Infection with a novel H10N8 influenza virus in humans was first described in China in December 2013, which raised concerns related to public health. This novel virus was subsequently confirmed to have originated from a live poultry market. However, whether this virus can infect other mammals remains unclear. In the present study, antibody specific for H10N8 influenza virus was detected in swine herds in southern China during serological monitoring for swine influenza virus. The pathogenicity and transmissibility of this H10N8 influenza virus to swine was examined. The results showed that swine are susceptible to infection with human-origin H10N8 influenza virus, which causes viral shedding, severe tissue lesions, and seroconversion, while infection with avian-origin H10N8 influenza virus causes only seroconversion and no viral shedding. Importantly, human-origin H10N8 influenza virus can inefficiently be transmitted between swine and cause seroconversion through direct contact. This study provides a new perspective regarding the ecology of H10N8 influenza virus and highlights the importance of epidemiological monitoring of the H10N8 influenza virus in different animal species, which will be helpful for preventing and controlling future infections by this virus.

## Introduction

Influenza A viruses (IAVs) are enveloped, segmented, and negative-sense RNA viruses of the family *Orthomyxoviridae* [1,2]. IAVs have a broad host range and can infect birds, humans, and other mammals, including swine, canine, feline, and horses, among others [3]. IAVs can be classified into different subtypes based on the antigenicity of the two major surface glycoproteins, haemagglutinin (HA) and neuraminidase (NA) [2,4]. Currently, 16 HA (H1–H16) and nine NA (N1–N9) subtypes have been identified in wild aquatic birds [5], and another two HA and NA subtypes (H17, H18 and N10, N11) have been identified in bats [6]. However, only three subtypes (H1N1, H2N2, and H3N2) are known to have established sustained human infections and circulate widely in humans. Of these three subtypes, H1N1 and H3N2 are currently responsible for causing seasonal influenza virus epidemics [7]. In addition to these three subtypes, many other IAV subtypes have been reported to cross the species barrier to infect humans, including H5N1, H5N6, H7N9, and H7N4 [8–11]. In December 2013, China reported the first human infection with a H10N8 avian influenza virus in Jiangxi Province, with two of the three infections resulting in death [12], raising concerns regarding the effects of H10N8 influenza viruses on public health. In China, H10N8 influenza viruses were previously isolated from the environment of Dongting Lake in Hunan Province in 2007 [13] and from a duck in a live poultry market (LPM) in Guangdong Province in 2012 [14], but it was unknown at that time if these strains could infect humans or other mammals. Several studies subsequently confirmed that the human H10N8 influenza virus originated from chickens in LPMs [15,16]. Additionally, a specific antibody for H10N8 influenza virus was detected in sera samples collected from animal workers and feral dogs at LPMs in Guangdong Province before the first H10N8 infection cases were recognized [17,18]. Interspecies transmission of avian influenza viruses to humans and other mammals has been consistently reported in the last two decades. Swine are susceptible to infection with both human and avian influenza viruses. Influenza virus may undergo reassortment to generate novel reassorted viruses in swine [19]; thus, swine are considered as “mixing vessels” in influenza ecology. Considering this important role, we conducted serological monitoring to detect H10N8 infection in swine herds. Eight serum samples were positive for H10N8 influenza virus based on hemagglutination inhibition (HI) and microneutralization (MN) assays during serological surveys in 2016–2017 in southern China. However, the pathogenicity and transmissibility of H10N8 influenza virus in swine remains unknown. Based on serological evidence, pathogenicity and transmission analyses of H10N8 influenza virus in swine were conducted in this study, which will be helpful for preventing future H10N8 infections and provide new perspectives regarding the transmission of H10N8 influenza virus.

## Materials and methods

### Viruses and serum samples

Human-origin H10N8 influenza virus strain A/Jiangxi-Donghu/346-1/2013 (JX346) and duck-origin strain A/duck/Guangdong/E1/2012 (GD-E1) were kindly provided by Professor Ming Liao and Professor Wenbao Qi, College of Veterinary Medicine, South China Agricultural University. Swine influenza virus A/swine/Guangdong/SS1/2012 (H1N1 Eurasian avian-like lineage) and A/swine/Guangdong/L22/2010 (H3N2) were isolated and preserved in our lab. Viruses were propagated in 9- to 11-day-old specific pathogen-free embryonated chicken eggs and stored at −80°C until use. A total of 2050 serum samples were collected from swine farms that were non-immunized for swine influenza virus from July 2016 to June 2017 in southern China.

### Serological survey of H10N8 infection in swine

Both the HI and MN assays were performed according to standard protocols provided by the World Health Organization. For the serological survey, all serum samples were treated with sialic acid receptor-destroying enzyme to remove nonspecific inhibition factors, and then tested by HI assay against strain JX346 using fresh chicken red blood cells. Samples with HI titers ≥ 1:40 were further evaluated by MN assay against JX346. In addition, HI and MN assays against GD-E1 were also performed for the serum samples that positive to JX346. The positive samples were also subjected to HI and MN assays against H1N1 and H3N2 swine influenza viruses. In this serological study, serum samples with HI titers < 1:40 were considered to be negative, while samples with HI titers ≥ 1:40 were considered as being infected by H10N8 virus; only samples with both HI and MN titers ≥ 1:40 were considered as having previously been infected with H10N8 virus.

### Animals and animal experiment

The animal experiments were approved by the Committee of Experiment Animal Welfare Ethical of South China Agriculture University. Nineteen one-month-old pigs were purchased from farms that were negative for porcine reproductive and respiratory syndrome virus and pseudorabies virus. They were confirmed to be free from influenza virus infection and were found to be seronegative for H1N1 and H3N2 swine influenza viruses by HI assay. The 19 pigs were randomly allocated into three groups (eight in group A, eight in group B, and three in group C). Five pigs in group A (A1–A5) and group B (B1–B5) were inoculated with JX346 and GD-E1 at a titer of 10^6^ TCID_50_/mL intratracheally (1 mL) and intranasally (1 mL), respectively. Three contact healthy pigs were commingled with infected pigs at two days post-infection (dpi) to investigate viral transmission in group A (A6–A8) and group B (B6–B8). Three control pigs in group C were inoculated intratracheally (1 mL) and intranasally (1 mL) with phosphate-buffered saline as a control group. Body temperatures and clinical signs were recorded daily, and nasal swabs were collected daily on 1–14 dpi for inoculated pigs and on 1–14 days post-contact (dpc) for contact pigs. Sera were collected at 7 dpi/dpc and 14 dpi/dpc for the HI and MN assays with homologous virus for each group to determine the antibody titer. One inoculated pig from each group (A5, B4, and C3) was necropsied at 3 dpi. Lung and tracheal tissues were collected for virus titer and pathology analysis. The viral titers from nasal swabs and lung samples were determined using Madin-Darby canine kidney (MDCK) cells in 96-well plates by the 50% tissue culture infective dose (TCID_50_) assay.

## Results

### Detection of H10N8-specific antibodies in swine herds

In the serological survey, a total of 2050 serum samples collected from swine farms in southern China were tested by HI assay against H10N8 strain JX346. A total of eight (8/2050) serum samples collected from four farms tested positive (HI titers of ≥1:40) for JX346, with HI titers ranging from 1:40 to 1:160 (Table 1). To confirm the results of the HI assay, an MN assay was performed for the eight HI-positive samples. The MN assay results showed that seven of the eight serum samples had MN titers ≥ 1:40 against JX346, with the highest MN titer reaching 1:640 (Table 1). HI and MN assays against GD-E1 were also conducted for these eight positive serum samples, and the result showed that these samples were also positive for GD-E1 based on HI and MN assay, and had similar HI and MN titers against JX346 (Table 1). Additionally, to exclude the possibility of cross-reactions with other swine influenza viruses, HI and MN assays for the H1N1 and H3N2 swine influenza viruses were conducted for the eight positive serum samples. The results showed that all samples were negative for H1N1 and H3N2 swine influenza virus (HI and MN titer < 1:10, Table 1).

**Table 1.** Results of serological assay for serum samples positive for H10N8 influenza virus.

Farm	Collection date	Sample name	H10N8		H1N1		H3N2	
			JX346 (HI/MN titer)	GD-E1 (HI/MN titer)	HI titer	MN titer	HI titer	MN titer
Farm A	12/2016	CZ1	**1:40/1:80**	**1:40/1:40**	<1:10	<1:10	<1:10	<1:10
		CZ2	**1:80/1:320**	**1:40/1:320**	<1:10	<1:10	<1:10	<1:10
		CZ3	**1:80/1:160**	**1:80/1:320**	<1:10	<1:10	<1:10	<1:10
Farm B	12/2016	MS1	**1:40/**1:20	**1:40**/**1:40**	<1:10	<1:10	<1:10	<1:10
		MS2	**1:40/1:40**	**1:40/1:80**	<1:10	<1:10	<1:10	<1:10
Farm C	2/2017	YC1	**1:40/1:80**	**1:40/1:160**	<1:10	<1:10	<1:10	<1:10
		YC2	**1:160/1:640**	**1:160/1:640**	<1:10	<1:10	<1:10	<1:10
Farm D	3/2017	HD	**1:80/1:320**	**1:40/1:160**	<1:10	<1:10	<1:10	<1:10

Note: Strains used in serological assay, A/Jiangxi-Donghu/346-1/2013 (H10N8), A/duck/Guangdong/E1/2012 (GD-E1), A/swine/Guangdong/SS1/2012 (H1N1), and A/swine/Guangdong/L22/2010 (H3N2).

HI, hemagglutination inhibition assay; MN, microneutralization assay.

### Infectivity of H10N8 influenza virus in swine

**Figure 1.**
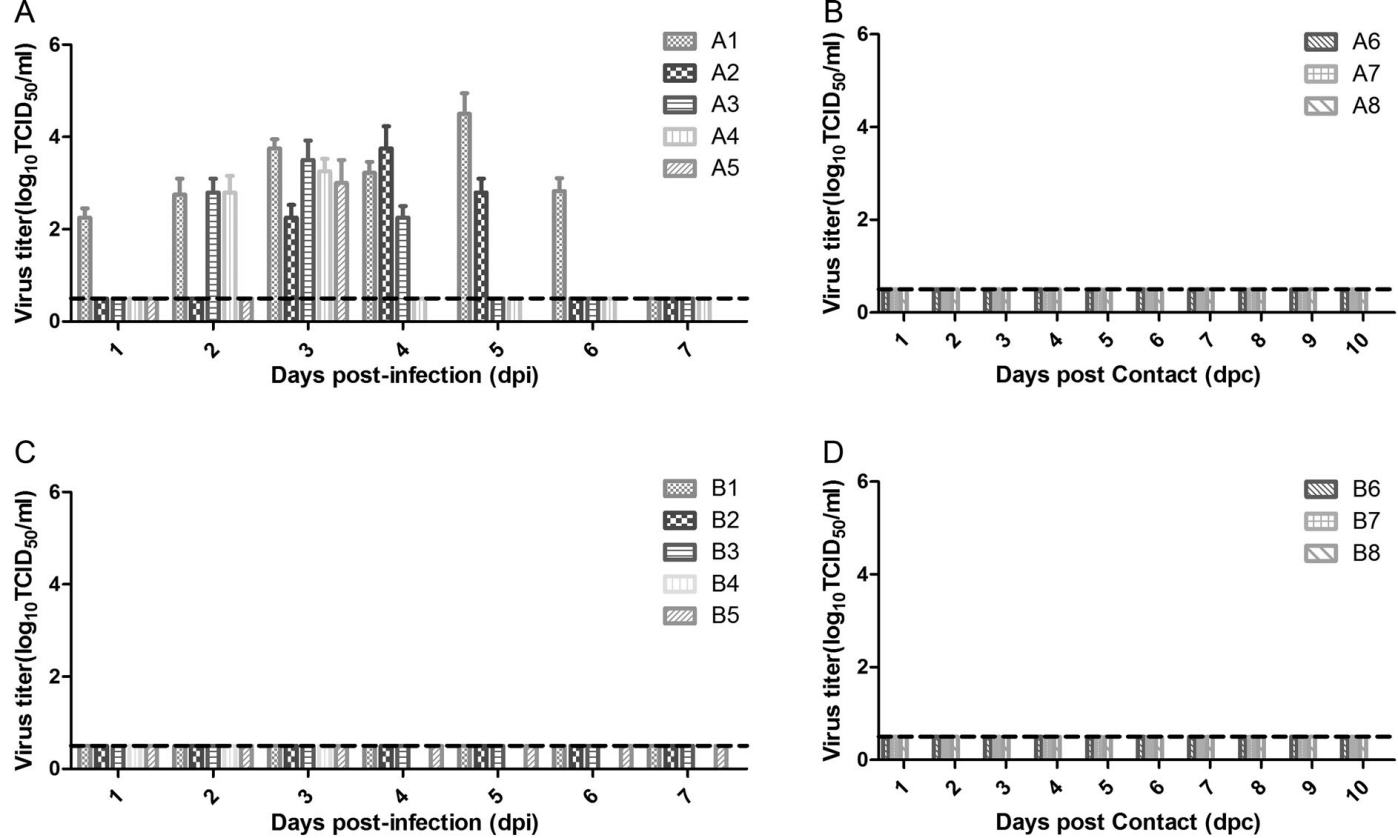
Virus shedding from nasal swabs in different groups. (A) JX346 inoculated group, (B) JX346 contact group, (C) GD-E1 inoculated group, and (D) GD-E1 contact group. Nasal swabs were collected from inoculated and contact pigs in different groups at 1–14 dpi/dpc, and viral titers were determined as the TCID_50_ in MDCK cells; no virus was detected after 8 dpi. The dotted lines indicate the lower limit of detection.

To investigate whether H10N8 influenza virus can infect swine, two groups of pigs were intratracheally and intranasally inoculated with JX346 or GD-E1 at a titer of 10^6^ TCID_50_/mL, respectively. All five pigs in group A infected with JX346 developed a fever at 2 days post-infection (dpi), which persisted for 2–3 days, and 2 of the 5 pigs developed clinical signs of infection (running nose). No pigs in group B infected with GD-E1 or control pigs in group C exhibited fever or any other clinical signs. Virus shedding was detected from the nasal swabs of pigs inoculated with JX346, which started as early as 1–3 dpi and lasted for 2–6 days, mostly peaking at 3–4 dpi (Figure 1(A)). The highest titer of virus shedding from nasal swabs was 10^4.5^ TCID_50_/mL in group A. However, no virus shedding from nasal swabs was detected in pigs inoculated with GD-E1 (Figure 1(C)). The viral titers of the lung and tracheal tissues collected from A5 and B4 at 3 dpi were determined; lung and tracheal tissues from A5 in the JX346 group showed titers of 10^4.75^ and 10^2.8^ TCID_50_/mL, respectively. However, no viral titers were detected in the lung and tracheal tissues from B4 in the GD-E1 group.

Sera were collected at 7 and 14 dpi to evaluate for antibody specific to H10N8 by HI and MN assay. All four pigs (one necropsied at 3 dpi) inoculated with JX346 in group A were seroconverted at 7 dpi, with the highest HI and MN titer reaching 1:320 and 1:1280, respectively, at 14 dpi (Table 2). Although pigs inoculated with GD-E1 in group B did not shed virus and showed no virus proliferation in the lung and trachea, two of the four pigs (one necropsied at 3 dpi) inoculated with GD-E1 in group B showed seroconversion at 7 dpi and one pig also seroconverted at 14 dpi (Table 2). The HI titer was obviously lower than that of group A at 14 dpi, MN titer also was detected at a range from 1:80 to 1:320 in group B (Table 2). Seroconversion was not observed in the control group. These results showed that swine can be infected with JX346, causing clinical signs, virus shedding, and seroconversion, with virus replication occurring in the lung and trachea tissues. In contrast, GD-E1 could not effectively infect swine and only induced seroconversion with low antibody levels. Thus, epidemiological monitoring of H10N8 influenza virus in swine herds should be further conducted to determine whether interspecies transmission of H10N8 influenza viruses has occurred in swine herds.

**Table 2.** Seroconversion and the HI and MN titer in pigs infected with H10N8 influenza virus.

Strain	Group	Pig	7 dpi/dpc		14 dpi/dpc	
			HI	MN	HI	MN
JX346	Inoculated	**A1**	**1:80**	**1:160**	**1:320**	**1:1280**
		**A2**	**1:40**	**1:40**	**1:80**	**1:640**
		**A3**	**1:40**	**1:80**	**1:160**	**1:1280**
		**A4**	**1:40**	1:20	**1:160**	**1:320**
	Physical contact	A6	<1:10	<1:10	<1:10	<1:10
		A7	<1:10	<1:10	**1:40**	**1:80**
		A8	<1:10	<1:10	<1:10	<1:10
GD-E1	Inoculated	**B1**	**1:40**	**1:80**	**1:80**	**1:320**
		**B2**	**1:40**	**1:160**	**1:40**	**1:320**
		**B3**	1:20	<1:10	**1:40**	**1:80**
		B5	<1:10	<1:10	<1:10	<1:10
	Physical contact	B6	<1:10	<1:10	<1:10	<1:10
		B7	<1:10	<1:10	<1:10	<1:10
		B8	<1:10	<1:10	<1:10	<1:10
PBS	Control	C1	<1:10	<1:10	<1:10	<1:10
		C2	<1:10	<1:10	<1:10	<1:10

Note: dpi, days post-infection; dpc, days post-contact.

### Transmissibility of H10N8 influenza virus in swine

To investigate whether H10N8 influenza virus can be transmitted between swine through physical contact, three pigs were commingled with infected pigs at 2 dpi in the JX346 and GD-E1 groups. No virus shedding was detected from the pigs in either group (Figure 1(B,D)), and no seroconversion was observed from contacted pigs in the GD-E1 group at 7 and 14 days post-contact (dpc) (Table 2). Interestingly, one contacted pig in the JX346 group was seroconverted at 14 dpc with an HI and MN titer of 1:40 and 1:80, respectively (Table 2), suggesting that this pig had contracted an H10N8 infection and further indicating that JX346 has the potential to be transmitted between pigs, though the efficiency is limited. These results show that avian-origin H10N8 virus cannot be transmitted between swine, while human-origin H10N8 virus could be transmitted between swine inefficiently by direct contact.

### Pathological changes of pigs infected with H10N8 influenza virus

**Figure 2.**
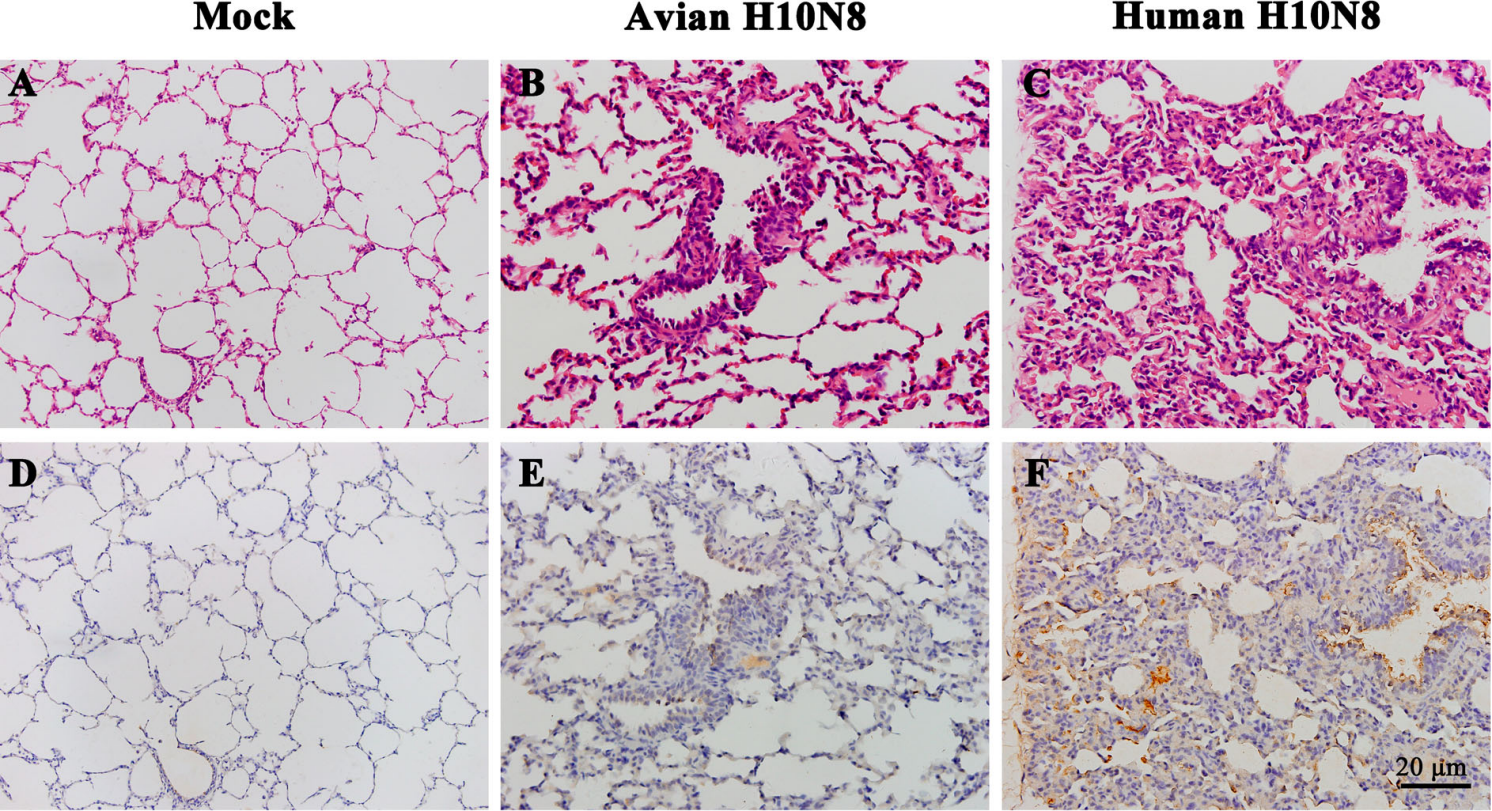
Pathological changes and viral replication in the lungs of inoculated pigs at 3 dpi. Hematoxylin and eosin staining (A–C) and immunohistochemical staining (D–E) of swine lungs. Pigs were inoculated with PBS (A and D), GD-E1 H10N8 virus (B and E), or JX346 H10N8 virus (C and F).

One inoculated pig from each group was necropsied at 3 dpi, and hematoxylin and eosin (HE) and immunohistochemical (IHC) staining of lung tissues was conducted. Microscopic observations of pulmonary sections revealed that swine infected with JX346 had obvious alveolitis and lesions in the lung tissue, including infiltration of inflammatory cells, widened alveolar walls, and degenerate and intact neutrophils within the alveolar lumen (Figure 2(C)). Swine infected with GD-E1 showed slight pathological changes in the lung tissue compared to the JX346 group; no severe tissue lesions were observed except for infiltration of inflammatory cells (Figure 2(B)). The IHC staining results revealed a large amount viral nucleoprotein (NP) in the JX346 group (Figure 2(F)), while no obviously viral protein was observed in the GD-E1 group (Figure 2(E)).

## Discussion

Rapid mutation and continuous reassortment are important features of influenza virus, which accelerate adaptation and interspecies transmission in new hosts. Numerous zoonotic influenza viruses that cause human infection have been documented in recent years, such as high-pathogenic avian influenza H5N1 infections in Hong Kong in 1997 [20], H1N1/2009 as a worldwide pandemic in 2009 [21], H7N9 avian influenza infection in China since 2013 [22], and novel reassorted H5N6 avian influenza virus infection in humans in 2014 [10]. In December 2013, human infection with H10N8 avian influenza virus was first reported in China, which resulted in three deaths [12], and H10N8 influenza virus infection of human origin from LPMs was subsequently confirmed by several research groups [15,16]. During serological monitoring of influenza virus in swine herds in 2016–2017 in southern China, we detected a specific antibody for H10N8 influenza virus for the first time and confirmed the result by MN assay. Serological analysis indicated that H10N8 influenza virus infection occurs in swine herds in nature. Su et al. also reported H10N8 influenza virus infections in feral dogs in LPMs before the first H10N8 infection cases were recognized [18]. These findings suggest that other mammals (such as swine and dog) are potential hosts of H10N8 influenza viruses. However, it is important to identify the origin of the H10N8 virus that caused seropositivity in these pigs. Based on previous studies on H10N8 strains, the most likely source of the virus is an avian species. Firstly, human infection with H10N8 has been confirmed to have originated in poultry from LPMs [15,16], with patients having visited LPMs and being exposed to the poultry a few days before the onset of illness. On the other hand, animal workers occupationally exposed to poultry may be at high risk of H10N8 infection, and antibodies against H10N8 virus among animal workers have been documented before the first human H10N8 case recognized [17]. Furthermore, there have been reports that H10N8 was circulating among poultry in southern China at the time of the prior studies [14,23], which provide a possible source of the swine infections. However, based on present study, avian-origin H10N8 virus can only ineffectively infect swine. It is unknown whether these viruses have undergone adaptative mutations during the infection process as has been seen in human infections.

We further investigated the pathogenesis of H10N8 influenza virus in swine, which showed that the human-origin strain caused more severe lesions than did the avian-origin strain. Many studies have shown that lysine at position 627 in PB2 is critical for virus pathogenicity and mammalian adaptation of avian influenza virus [24–26]. JX346 contained the 87E, 292 V, 340 K, 588 V, and 627 K mutations in the PB2 gene; these mutations have been reported to play a critical role in mammalian adaptation of human-origin H10N8 influenza virus [27]. These mutations may be responsible for the differences in pathogenesis between human and avian-origin strains in swine.

Swine have both sialic acid (SA) receptors (SA α-2,3-galactose and SA α-2,6-galactose) present in the respiratory tract; thus, swine are susceptible to infection with both human and avian influenza virus [19]. In contrast, the human respiratory tract mainly contains the SA α-2,6-galactose receptor [28]. Several studies have reported that human-origin H10N8 influenza viruses shows a strong preference for the avian receptor [29–31], indicating that the current human-origin H10N8 isolates are poorly adapted for human-to-human transmission. However, the human-origin H10N8 strain was inefficiently transmitted between swine through direct contact in this study. Notably, whether H10N8 acquires adaptive mutations and if the receptor binding properties are altered during virus evolution remains unclear; thus, continuous epidemiological monitoring of H10N8 influenza virus in different animal species (such as avian and swine) should be further conducted.

However, there are several limitations in evaluating the transmissibility of JX346 between swine in this study. Firstly, no virus could be detected in the transmission group. The only evidence for transmission is based on serological assay, and the HI and MN titer is lower compared with the infected group. Secondly, the number of animals used in the transmission experiment is not sufficient and only one pig had titers above the cut-off for HI and MN, which limits our understanding of the transmissibility of JX346 in swine.

In summary, we detected a specific antibody for H10N8 influenza virus in swine herds in southern China for the first time, and further confirmed that human-origin H10N8 strain could infect swine, causing clinical signs, virus shedding, and seroconversion. More importantly, human isolates could be transmitted between swine inefficiently through direct contact. This study provides a new perspective regarding the ecology of H10N8 influenza virus and highlights the importance of epidemiological monitoring of influenza viruses in different animal species, which will be helpful for implementing prevention and control measures.
